# Patellar tendon morphology in trans-tibial amputees utilizing a prosthesis with a patellar-tendon-bearing feature

**DOI:** 10.1038/s41598-019-52747-9

**Published:** 2019-11-08

**Authors:** Kai-Yu Ho, Michelle Harty, Jessica Kellogg, Kelly Teter, Szu-Ping Lee, Yu-Jen Chang, Gregory Bashford

**Affiliations:** 1 Department of Physical Therapy, University of Nevada, Las Vegas, 4505 S. Maryland Pkwy, Las Vegas, NV 89154 USA; 2 Division of Physical Therapy, West Virginia University, 64 Medical Center Drive, P.O. Box 9226, Morgantown, WV 26506 USA; 3 Department of Biological Systems Engineering, University of Nebraska-Lincoln, 230 L. W. Chase Hall, P. O. Box 830726, Lincoln, NE 68583 USA

**Keywords:** Orthopaedics, Medical imaging, Tendons

## Abstract

A patellar-tendon-bearing (PTB) bar is a common design feature used in the socket of trans-tibial prostheses to place load on the pressure-tolerant tissue. As the patellar tendon in the residual limb is subjected to the perpendicular compressive force not commonly experienced in normal tendons, it is possible for tendon degeneration to occur over time. The purpose of this study was to compare patellar tendon morphology and neovascularity between the residual and intact limbs in trans-tibial amputees and healthy controls. Fifteen unilateral trans-tibial amputees who utilized a prosthesis with a PTB feature and 15 age- and sex- matched controls participated. Sonography was performed at the proximal, mid-, and distal portions of each patellar tendon. One-way ANOVAs were conducted to compare thickness and collagen fiber organization and a chi-square analysis was used to compare the presence of neovascularity between the three tendon groups. Compared to healthy controls, both tendons in the amputees exhibited increased thickness at the mid- and distal portions and a higher degree of collagen fiber disorganization. Furthermore, neovascularity was more common in the tendon of the residual limb. Our results suggest that the use of a prosthesis with a PTB feature contributes to morphological changes in bilateral patellar tendons.

## Introduction

There are an estimated 185,000 new amputations each year in the United States and 65% of persons living with limb loss had an amputation of the lower extremity, of which trans-tibial amputation accounts for more than 50%^1^. After a trans-tibial amputation procedure, qualified amputees are fitted with a prosthesis allowing them to perform daily activities^2^. The patellar-tendon-bearing (PTB) bar is a common and functional prosthetic socket design feature used in individuals with trans-tibial amputation^3^. This design takes advantage of the patellar tendon as a weight-bearing structure due to its pressure tolerance and reduces loading to more pressure sensitive areas of the residual limb^3^. To bear weight, the prosthetic socket is designed with a convex contour that directs compressive forces to the patellar tendon region during weight- bearing activities^4^. This loading pattern is distinctly different from the tensile force a patellar tendon typically experiences during weight-bearing^5^. In this situation the patellar tendon of the residual limb experiences constant or intermittent perpendicular compression from the prosthetic socket as well as the typical tensile load^4^.

As the patellar tendon of the residual limb is subjected to a different loading condition, it is possible that tendon remodeling and degeneration can occur over time in trans-tibial amputees using prostheses with a PTB design. In animal models, abnormal perpendicular forces applied to tendons have been found to cause morphological changes similar to those observed in a degenerative tendon^6^. These changes include collagen fiber disorganization, increased water content, increased glycosaminoglycan content, thinner collagen fibers, reduced overall collagen content, increased type II collagen, and reduced tendon stiffness^6^.

Using ultrasound imaging, several previous studies on non-amputees have demonstrated that chronic overloading of the patellar tendon causes gross morphological changes (increased tendon thickness^7,8^), intra-tendinous morphological changes (increased collagen fiber disorganization^8^), and neovascularity^9,10^. Although these patellar tendon morphological abnormalities may develop in amputees using a prosthesis with a PTB feature, there is limited evidence to support a correlation. To date, only one preliminary study revealed that the patellar tendon is thicker in the residual limb of trans-tibial amputees^11^. However, there have been no comparisons of neovascularity and intra-tendinous and gross morphological changes in the patellar tendon between trans-tibial amputees using a PTB design and healthy controls. Therefore, the purpose of this study was to use sonography to compare patellar tendon morphology and neovascularity between the residual and intact limbs in unilateral trans-tibial amputees using a prosthesis with a PTB feature and healthy controls.

## Methods

### Participants

Fifteen participants with trans-tibial amputation and 15 healthy controls participated (Table 1). Inclusion criteria for participants with unilateral trans-tibial amputation included (1) at least 18 years old, (2) had used a prosthesis with a PTB feature for a minimum of one year prior to the study, and (3) had utilized a prosthesis with a PTB feature for at least one hour of combined weight bearing (e.g. standing, walking, running, lower extremity weight-bearing resistance training) per day. Healthy controls were sex- and age-matched (within 10% difference) to their amputee-counterparts and had at least one hour of combined weight bearing per day. Participants in the control group were excluded if they reported pain in the patellar tendon. In addition, participants were excluded from the study if they were non-ambulatory. The data from an existing study was used to estimate the sample size for detecting changes in tendon morphology between normal and degenerative tendons^8^. With 95% power, an α level of 0.05, and a calculated effect size of 1.7, 11 participants would be needed to detect a difference in tendon morphology between healthy and injured tendons. However, 15 participants in each group were recruited due to the exploratory nature of this study. This study was approved by the Institutional Review Boards at the University of Nevada, Las Vegas (IRB # 1080294) and was performed in accordance with the relevant guidelines/regulations. All participants provided written informed consent prior to data collection, per Institutional Review Board protocols.

**Table 1:** Participants Characteristics.

Gender	Trans-tibial amputees (n = 15)	Healthy controls (n = 15)	P value
	3 Females; 12 Males	3 Females; 12 Males	
Age, y	52.5 ± 19.1	51.4 ± 17.7	0.868
Height, cm	180.2 ± 9.1	176.3 ± 6.9	0.196
Weight, kg	93.0 ± 20.4	81.0 ± 13.0	0.064
Body mass index (BMI), kg/m^2^	28.7 ± 6.0	25.9 ± 2.8	0.114
VISA-P	70.3 ± 20.2	94.0 ± 5.4	0.000*
Side of amputation	7 Right, 8 Left	NA	NA
Years wearing prosthesis	15.9 ± 17.4	NA	NA
Reason for amputation	2 infection; 3 peripheral artery disease; 9 trauma; 1 congenital defects with subsequent amputation	NA	NA
PEQ (Pain in residual limb)	68.5 ± 38.9	NA	NA
PEQ (Ambulation)	80.3 ± 30.6	NA	NA
PLUS-M T score	62.9 ± 11.3	NA	NA

*Indicates a statistically significant difference between trans-tibial amputees and healthy controls using an independent t test.

### Instrumentation

High-resolution ultrasound images were acquired using a commercial ultrasound system (GE LOGIQ-e, GE Healthcare, Milwaukee, WI, USA) with its musculoskeletal knee preset. Brightness-mode images and power Doppler images were captured using a linear array transducer (GE 12L-RS, bandwidth 5–13 MHz, width 38.4 mm) at a central frequency of 10 MHz and depth of 2 cm.

### Procedures

Data collection occurred in two consecutive phases. In the first phase, participants’ weight and height were measured. Participants with trans-tibial amputation were then asked to fill out the following questionnaires: Victorian Institute of Sport Assessment-Patellar Tendon (VISA-P)^12^, Prosthetic Evaluation Questionnaire (PEQ)^13^, and Prosthetic Limb Users Survey of Mobility (PLUS-M)^14^. Healthy controls were asked to fill out the VISA-P survey. The VISA-P is a reliable survey that evaluates knee pain/function specifically related to patellar tendon pathology^15–17^. The score of VISA-P ranges from 0 to 100 with lower scores indicating worse pain/function and a score lower than 80 is commonly used for the diagnosis of patellar tendinopathy^16,17^. The PEQ measures various domains of lower limb prosthesis use (e.g., ambulation, pain, and appearance) and has been found to be reliable^13^. The score range for each PEQ domain is from 0 and 100 with lower values indicating a worse outcome^13^. The PLUS-M is a valid, self-report instrument for measuring mobility in adults using lower limb prosthesis^14^. The PLUS-M T-score ranges from 21.8 to 71.4 and a higher score indicates better mobility^14^.

Immediately following survey completion, ultrasound imaging was used to obtain images of each participant’s patellar tendons on both limbs. Participants were seated with knees and hips flexed to 90° on the edge of a treatment table^8,18,19^. While this testing position may place tension to the patellar tendon, it is deemed a reliable setup for examining gross and intra-tendinous morphology of the patellar tendon^8,19^. The investigator palpated and marked the tendon-patella junction (proximal portion) and tendon-tibial tuberosity junction (distal portion), and identified the mid-point between the two bone-tendon junctions (mid-portion). To obtain tendon morphology, longitudinal brightness-mode images were acquired at the proximal, mid-, and distal portions of the patellar tendon. To identify neovascularity, tendons were assessed at the same three locations using power Doppler imaging (gain = 25) and images were captured if neovascularization was observed. Neovascularity was defined as visual evidence of red, pulsatile coloration indicating blood flow within the boundaries of the tendon^20^. To optimize the reliability and accuracy for the measurements of tendon morphology and neovascularity, all image acquisition was performed by the same trained investigator who applied minimal pressure to the patellar tendon during ultrasound imaging examination^20^.

### Data analysis

**Figure 1:**
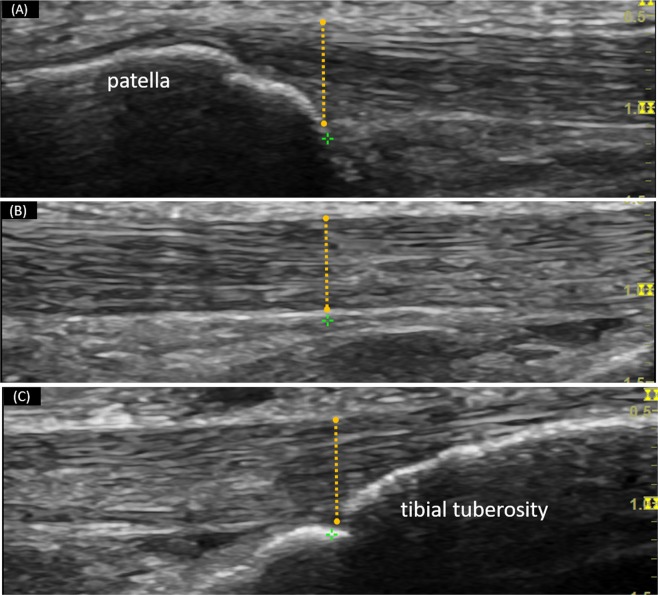
Measurement for tendon thickness: the distance between the borders of the patellar tendon was manually identified and quantified at the (**A**) proximal, (**B**) mid-, and (**C**) distal portions of the patellar tendon.

All images were exported in JPEG format to a personal computer for the analyses of tendon thickness using ImageJ software (National Institutes of Health, Bethesda, MD, USA). The tendon thickness was measured on each longitudinal image (i.e., proximal, mid-, and distal portions), which was defined as the perpendicular distance between the borders that outline the patellar tendon (Fig. 1). Specifically, the thickness of the proximal portion was measured at the intersection of patellar tendon and the apex of the patella (Fig. 1A)^21,22^. The distal thickness was measured at the patellar tendon-tibial tuberosity intersection (Fig. 1C)^23^. the For mid-potion of the patellar tendon, the thickness was measured at the center of the mid-portion ultrasound image (mid-point between the tendon-patella and tendon-tibial tuberosity junctions) (Fig. 1B)^21^.

**Figure 2:**
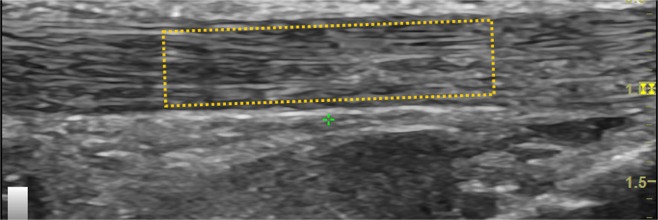
Measurement for peak spatial frequency radius (PSFR) of the patellar tendon: a region of interest (ROI) in the middle 50% of the tendon was manually outlined and analyzed.

For analyses of the intra-tendinous morphology, a custom MATLAB program (Mathworks, Natick, MA, USA) was used to quantify collagen fiber organization on the longitudinal image of the mid-portion tendon. A region of interest (ROI) in the middle 50% of the tendon was manually outlined for the calculation of intra-tendinous morphology (Fig. 2). Within the ROI, 32 × 32 pixel kernels were extracted and all possible kernels were processed with a two-dimensional Fast Fourier Transform, from which the peak spatial frequency radius (PSFR) parameter was extracted. The intra-tendinous morphology analysis process has previously been described in full^19,24^. Typically, a lower PSFR reflects greater collagenous disarray, which is one underlying structural phenomenon of degeneration^25^. A higher PSFR reflects a greater collagenous density, which is associated with greater stiffness and elastic modulus^24,26^.

### Measurement reliability

To establish intra-rater reliability of tendon morphological measures (i.e., thickness and PSFR), the investigators performed repeated measurements from five participants on two separate days, seven days apart. Intraclass correlation coefficients (ICCs) and standard errors of measurements (SEMs) were calculated. The﻿ investigators showed excellent measurement reliability and low SEMs for PSFR (ICC = 0.998; SEM = 0.00646 mm^−1^) and thickness (ICC = 0.984; SEM = 0.037 mm).

### Statistical analyses

For participants with trans-tibial amputation, the patellar tendon morphological measurements of both limbs (residual and intact) were analyzed statistically. For healthy controls, tendon morphological data of the side matching the residual limb of their amputee counterparts was used for statistical analyses. One-way ANOVAs and post-hoc analyses were conducted to compare patellar tendon thickness and collagen fiber organization between the three tendon groups (residual, intact, and control). A chi-square analysis was used to compare the frequency of the presence of neovascularity in the three tendon groups. All statistical analyses were performed with SPSS Statistics 24 for Windows (International Business Machines Corp, Armonk, NY, USA). *A priori* alpha was set at 0.05.

## Results

### Participant characteristics

The participant characteristics are shown in Table 1. Both groups had similar age, height, weight, body mass index (BMI), and sex proportion. However, the amputee participants had a significantly worse pain/function related to patellar tendon, evidenced by a lower VISA-P score (p < 0.001). Additionally, the average VISA-P score for healthy controls was 94 with the lowers score being 85, which indicated that the healthy cohort’s patellar tendon was fairly healthy^16,17^. For participants with trans-tibial amputation, the main cause of amputation was trauma (60%), followed by peripheral artery disease (20%), infection (13%), and congenital defects (7%). The average years of wearing prosthesis was 15.9 years (1–50 years).

### Gross morphology: tendon thickness

**Figure 3:**
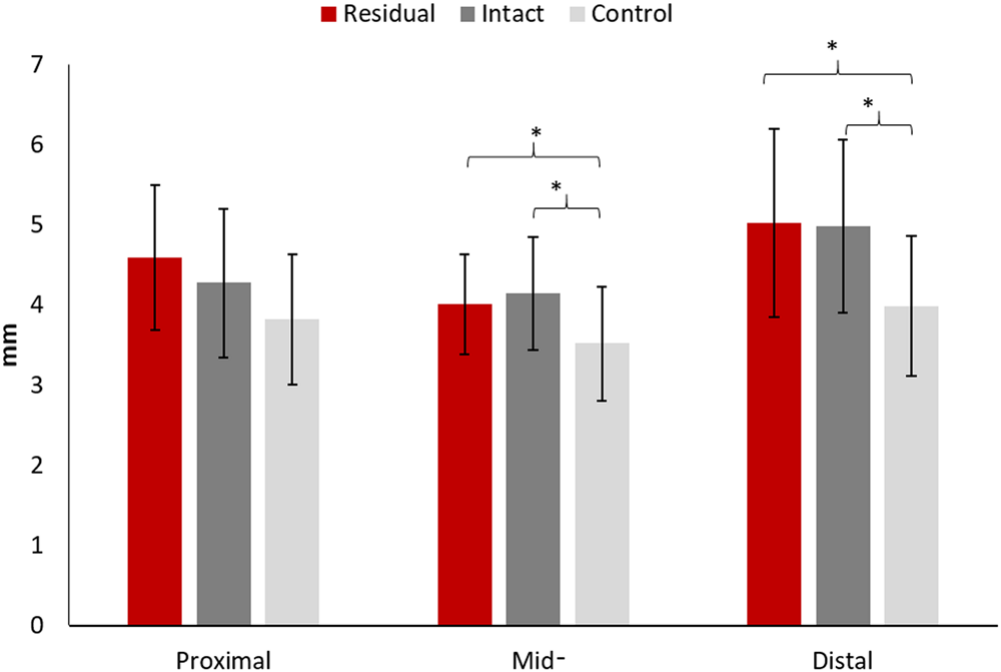
The comparisons of patellar tendon thickness between the residual and intact limbs in trans-tibial amputees and healthy controls’ limbs. *Indicates a statistically significant difference from the control limb from ANOVA and post-hoc analyses.

One-way ANOVAs revealed a statistically significant difference in the thickness at the mid- (p = 0.038) and distal (p = 0.014) portions of the patellar tendon. Compared to the tendons of healthy controls, the post-hoc analyses revealed that the patellar tendons were significantly thicker in both the residual and intact limbs of the trans-tibial amputees at the mid- (residual = 4.0 ± 0.6 mm vs. control = 3.5 ± 0.7 mm, p = 0.048; intact = 4.1 ± 0.7 mm vs. control = 3.5 ± 0.7 mm, p = 0.016) and distal (residual = 5.0 ± 1.2 mm vs. control = 4.0 ± 0.9 mm, p = 0.010; intact = 5.0 ± 1.1 mm vs. control = 4.0 ± 0.9 mm, p = 0.013) portions. The tendon thickness differences in the mid- and distal portions between the residual and intact limbs were not significant (mid-portion: p = 0.603 and distal portion: p = 0.916). In addition, the ANOVA revealed no statistically significant difference in proximal tendon thickness between the three tendon groups (p = 0.068) (Fig. 3).

### Intra-tendinous morphology: collagen fiber organization

**Figure 4:**
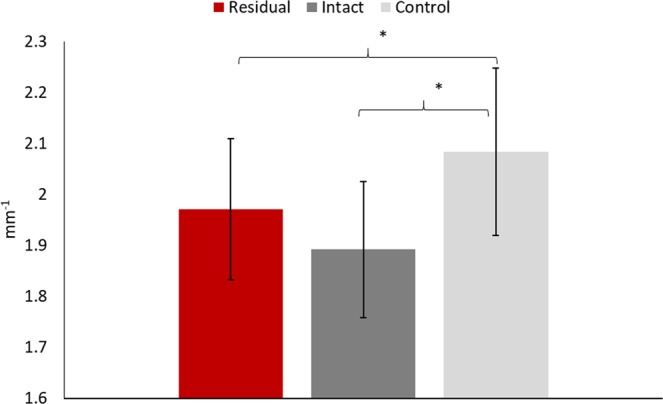
The comparisons of peak spatial frequency radius (PSFR) between the residual and intact limbs in trans-tibial amputees and healthy controls’ limbs. *Indicates a statistically significant difference from the control limb from ANOVA and post-hoc analyses.

There was a statistically significant difference in PSFR from one-way ANOVA (p = 0.003). Compared to the tendons in healthy controls, the post-hoc analyses revealed that the patellar tendons had a significant smaller PSFR in the residual and intact limbs of the trans-tibial amputees (residual = 1.97 ± 0.14 mm^−1^ vs. control = 2.08 ± 0.16 mm^−1^, p = 0.041; intact = 1.89 ± 0.13 mm^−1^ vs. control = 2.08 ± 0.16 mm^−1^, p = 0.001). The difference in tendon PSFR between the residual and intact limbs in amputees was not significant (p = 0.146) (Fig. 4).

### Neovascularity

Neovascularity was observed in eight tendons of the residual limbs (53.3%) and three tendons of the intact limbs (20.0%) in 15 participants with trans-tibial amputation. Neovascularity was not observed in any tendon of the healthy controls (0 out of 30 limbs [0%]). Chi-square revealed a statistically significant difference in the frequency of the presence of neovascularity between the three tendon groups (X^2^ = 19.035; DF = 2; p < 0.001). Post-hoc analyses, examined by adjusted residuals, revealed that the neovascularity of the patellar tendon was statistically significantly more common in the residual limb (adjusted residual = 5.3).

## Discussion

To the best of our knowledge, this is the first study to compare the morphological measures and neovascularity of the patellar tendon between the unilateral trans-tibial amputees and sex- and age-matched non-amputees. Our findings suggested that tendons in both legs of the amputee participants exhibited increased thickness at the mid- and distal portions and a higher degree of collagen fiber disorganization when compared to controls. Furthermore, neovascularity was more commonly observed in the patellar tendon of the residual limb.

From our data, it is speculated that both tendons in trans-tibial amputees might experience abnormal mechanical loading, which led to degenerative changes in gross and intra-tendinous morphology. In our study, the average PSFR values for the tendons in the residual and intact limbs were 1.97 mm^−1^ and 1.89 mm^−1^, respectively. These values were lower than the PSFR of healthy patellar tendons (2.20–2.30 mm^−1^) reported by Pearson *et al*.^18^ and that of healthy tendons (2.08 mm^−1^) in the current study. Furthermore, the higher thickness observed in the patellar tendon of residual and intact limbs may be indicative of tendon degeneration, as suggested in the existing literature^7,8^. One potential contributing factor leading to altered morphology of the tendons in the residual and intact limbs was that the amputee participants had a slightly higher body weight and BMI, even though the differences were not statistically different between amputee participants and healthy controls (Table 1). Tas *et al*.^27^ have reported that obesity (BMI > 25 kg/m^2^) is related to an increase in patellar tendon thickness in healthy sedentary individuals. Furthermore, it has been found that the intact limb of trans-tibial amputees exhibits a higher knee extensor moment during walking compared to the residual limb, which is thought to result in higher tensile loading of the patellar tendon in the intact limb^28^. Thus, while only the tendon of the residual limb experienced an abnormal perpendicular compressive force from the PTB feature, the speculated higher tensile loading experienced by the tendon of the intact limb during daily locomotion may cause morphological changes that are similar to those observed in the residual limb.

Additionally, the tendon of the residual limb in trans-tibial amputees using a PTB feature might develop more advanced degeneration, evidenced by a higher proportion of neovascularity in the tendon of residual limb. In our study, the observed proportion of neovascularity in the residual limb of trans-tibial amputees was higher than that reported in athletes with tendon degeneration (42%)^10^. The high incidence of neovascularity observed in the residual limb may be attributed to the abnormal perpendicular compressive loading from the PTB feature. Convery and Buis^29^ have demonstrated that the peak pressure to the patellar tendon load-bearing area in such designs can be in excess of 100 kPa, thereby damaging the focal tissues in this region. The high pressure from the PTB feature of the socket may also contribute to the localized tendon thickening, particularly in the mid- to distal portion of the patellar tendon.

With respect to the comparisons of tendon thickness in trans-tibial amputees between our work and existing literature, our findings did not agree with the results reported by Ozcakar *et al*.^11^ showing that the tendon thickness on the residual limb is greater than that of the intact limb. The inconsistent findings between our work and Ozcakar *et al*.’s study may be explained by different age (Ozcakar *et al*.: 28.6 ± 6.1 years vs. current work: 52.5 ± 19.1 years) and years of wearing prosthesis (Ozcakar *et al*.: 4.9 ± 6.1 years vs. current work: 15.9 ± 17.4 years) between the participants of the two studies. In our study, the similar tendon thickness observed in the residual and intact limbs may be related to the fact that both tendons of our amputee participants were subjected to abnormal mechanical loading for a longer period of time.

The present study has several limitations that should be recognized. First, although a lower PSFR value was observed in both tendons of residual and intact limbs, the exact cut-off PSFR value defining the abnormal tendon intra-tendinous morphology remains unknown. Nevertheless, the data presented in this study provides important information regarding the intra-tendinous morphological changes in the patellar tendon of unilateral trans-tibial amputees who use a prosthesis with a PTB feature. Second, most of our amputee participants had trauma as their cause of amputation (60%). As peripheral vascular disease is the primary cause of lower extremity amputation in general population (over 70%)^30^, our data may not be representative of the entire trans-tibial amputee population. Third, the participants’ knee was placed at 90° of flexion during the ultrasound imaging examination. This position might cause compression of small blood vessels^20^, thereby underestimating the rate of neovascularity observed in our participants. Thus, caution should be made when comparing our findings to those that are conducted at a less flexed knee position. Furthermore, while all participants were included only when they had at least one hour of weight bearing per day, we did not collect specific data regarding participant’s physical activity level during daily living. Therefore, it is unclear if the tendon morphological data reported in this study was affected by participant’s physical activity level. As trans-tibial amputees are often reported with a lower physical activity level when compared with healthy individuals^31^, the suspected reduced accumulative limb loading from lower physical activity level might somewhat be counterbalanced by increased loading resulting from a higher body weight observed in our amputee participants. Due to the fact that the tendon loading was not quantified in this study, future research should use a longitudinal design and more precise measure of tendon loading to examine the effect of chronic loading on patellar tendon morphology.

In conclusion, we observed that both tendons in the amputee participants exhibited increased thickness at the mid- and distal portions and a higher degree of collagen fiber disorganization when compared to healthy controls. Furthermore, neovascularity was more commonly observed in the tendon of residual limb. Our results suggest that the use of a prosthesis with a PTB feature contributes to degenerative, morphological changes in bilateral patellar tendons and neovascularity in the residual tendon of unilateral trans-tibial amputees.
